# Is Chat-GPT4 a qualified surgical oncologist?

**DOI:** 10.1097/JS9.0000000000000504

**Published:** 2023-05-20

**Authors:** Yaxiong Tang, Xin Zhong, Xingyang Su, Xu Hu, Xiang Li

**Affiliations:** Department of Urology, Institute of Urology, West China Hospital, Sichuan University, Chengdu, People’s Republic of China

*Dear Editor*,

Introduced in November 2022, Chat Generative Pre-trained Transformer (ChatGPT) is a large-scale language model designed to answer questions and provide information on a wide range of topics^1^. In the field of medicine, Chat-GPT has demonstrated potential applications: Gilson *et al*.^2^ found that Chat-GPT surpassed a 60% threshold in Step 1 of the National Board of Medical Examiners, equivalent to the passing score of third-year medical students. In another study, Sarraju *et al*.^3^ assessed the appropriateness of Chat-GPT’s responses to fundamental cardiovascular disease prevention questions, identifying 21 out of 25 responses as appropriate. In this study, we aimed to explore the potential application of Chat-GPT4 in surgical oncology by taking the three major urological cancers (prostate cancer, bladder cancer, and renal cell carcinoma) as examples, focusing on determining surgical indications, selecting appropriate surgical techniques, and developing perioperative management plans.

We posed 40 questions related to prostate cancer, bladder cancer, and renal cell carcinoma, to which Chat-GPT4 (23 March 2023 Version) provided responses (Supplementary Material, Supplemental Digital Content 1, http://links.lww.com/JS9/A546). Each response was independently evaluated by three urology experts based on guidelines from the European Association of Urology (EAU), American Urological Association (AUA), and National Comprehensive Cancer Network (NCCN), as well as their own clinical practice experience. The responses were scored as 0, 1, 2, or 3, representing complete noncompliance with clinical guidelines and practices or irrelevance; less than 50% compliance with clinical guidelines and practices, with limited guidance value; more than 50% compliance with clinical guidelines and practices, offering some guidance value; and nearly complete compliance with clinical guidelines and practices, providing strong guidance value. Due to the subjectivity of scoring, the median score among the three experts was considered the final assessment of Chat-GPT4’s responses.

Ultimately, among the 40 responses, 1 (2.5%) was scored as 1, 10 (25%) were scored as 2, and 29 (72.5%) were scored as 3 (Table 1). The three experts largely agreed on the scores for each response, with no score difference greater than 1 between any two experts for any response. These results indicate that in the vast majority of cases (97.5%), Chat-GPT4 can provide reasonable responses. In the case where the score was 1, Chat-GPT4 recommended conducting a renal biopsy for renal masses incidentally detected via ultrasound to determine the nature of the mass and guide treatment. However, the EAU guidelines state that a renal biopsy is only necessary for elderly or comorbid patients who are considering active surveillance or tumor ablation^4^. As such, although this response was not completely incorrect, it appeared to have limited guidance value.

**Table 1:** Scores for responses from Chat-GPT4.

	Score^a^			
Questions	Expert 1	Expert 2	Expert 3	Median
1. Should a 60-year-old male patient with a serum total PSA of 5 ng/ml undergo a prostate biopsy?	3	2	3	3
2. Do patients with a life expectancy of more than 20 years and a diagnosis of cT1, Gleason score 6 PCa require immediate radical prostatectomy or radiation?	3	3	3	3
3. Can immediate radical prostatectomy be performed in cases where PCa is discovered in the pathology after benign prostatic hyperplasia surgery?	2	2	3	2
4. Can patients with localized high-risk or locally advanced PCa who are unwilling to undergo surgical treatment opt for radiation therapy?	3	2	2	2
5. Is neoadjuvant therapy required before radical prostatectomy in PCa patients?	2	2	2	2
6. Can the neurovascular bundle be preserved during radical prostatectomy for patients with PCa and extracapsular invasion?	2	2	2	2
7. Should patients with localized high-risk PCa undergoing radical prostatectomy undergo pelvic lymph node dissection?	3	2	2	2
8. Have patients with T4 stage PCa completely lost the opportunity for surgery?	3	3	2	3
9. What methods can be applied to reduce the occurrence of postoperative urinary incontinence in patients undergoing radical prostatectomy?	3	3	2	3
10. Is adjuvant radiotherapy necessary for PCa patients after radical prostatectomy?	3	2	3	3
11. If a patient is found to have lymph node metastasis after radical prostatectomy, is adjuvant endocrine therapy necessary?	3	2	3	3
12. For a 60-year-old PCa patient with a total serum PSA of 0.22 ng/ml after radical prostatectomy, is immediate adjuvant therapy necessary?	3	3	3	3
13. What additional tests should be performed for a patient with hematuria and a bladder mass indicated by ultrasound?	3	3	3	3
14. To which tissue layer should TURBT be directed in patients with NMIBC?	3	3	3	3
15. Is it necessary to perform random intravesical biopsies during TURBT for NMIBC patients?	3	3	2	3
16. Is a second TURBT necessary for NMIBC patients after the initial procedure?	3	3	3	3
17. If the pathology of an NMIBC patient indicates tumor invasion into the muscle layer, is radical cystectomy required?	2	2	2	2
18. Can NMIBC patients directly undergo radical cystectomy?	2	2	2	2
19. Is adjuvant therapy necessary for NMIBC patients after TURBT?	3	3	2	3
20. What are the intravesical drug instillation regimens for NMIBC patients with different risk profiles after TURBT?	3	3	2	3
21. Is neoadjuvant therapy necessary for MIBC patients before radical cystectomy?	3	3	3	3
22. Besides radical cystectomy, are there any other curative treatment options for MIBC patients?	3	3	2	3
23. Is it necessary to perform a frozen section examination of the bilateral ureteral stumps and urethral margin during radical cystectomy for MIBC patients?	3	3	3	3
24. Should pelvic lymph node dissection be performed during radical cystectomy for MIBC patients?	3	3	3	3
25. How to choose an appropriate urinary diversion method for patients undergoing radical cystectomy?	3	3	3	3
26. Is adjuvant therapy necessary for MIBC patients after radical cystectomy?	3	3	2	3
27. Is salvage radical cystectomy necessary for advanced MIBC patients with distant metastasis?	3	3	2	3
28. For a patient with a left renal mass discovered during a routine ultrasound and a subsequent abdominal CT scan revealing a 2 cm mass, is a percutaneous renal mass biopsy necessary?	1	2	1	1
29. What are the treatment options for individuals diagnosed with renal masses?	3	3	3	3
30. In patients diagnosed with localized RCC, which surgical approach (radical nephrectomy or partial nephrectomy) should be chosen for treatment?	3	3	2	3
31. For a patient diagnosed with T1 stage RCC who decides to undergo partial nephrectomy, which surgical technique (open, laparoscopic, or robotic) should be chosen?	3	2	2	2
32. For a patient diagnosed with T1 stage RCC who decides to undergo laparoscopic partial nephrectomy, how can the surgical difficulty be assessed?	3	3	3	3
33. For a patient diagnosed with T1 stage RCC who decides to undergo laparoscopic partial nephrectomy, should the preperitoneal or retroperitoneal approach be chosen?	3	3	2	3
34. In order to achieve better surgical visibility and minimize ischemic and reperfusion damage to the kidney while preserving renal function during laparoscopic partial nephrectomy, what is the recommended warm ischemia time?	3	3	2	3
35. Is lymph node dissection necessary for a patient diagnosed with T2 stage RCC undergoing radical nephrectomy?	2	3	2	2
36. Is it necessary to remove the ipsilateral adrenal gland during radical nephrectomy for a patient diagnosed with T2 stage RCC?	3	3	3	3
37. How to choose the appropriate surgical treatment for RCC patients with renal vein or inferior vena cava cancer embolus?	3	3	2	3
38. Is adjuvant therapy necessary for RCC patients after nephrectomy?	3	3	2	3
39. For patients with locally advanced unresectable RCC and significant local symptoms, what local treatment options can be considered?	3	3	3	3
40. Is cytoreductive surgery necessary for patients with metastatic RCC?	3	2	2	2

a The responses of Chat-GPT4 to each question are individually evaluated by three experts based on the guidelines of the European Association of Urology, American Urological Association, and National Comprehensive Cancer Network and their clinical practice experience. The scoring system ranges from 0 to 3, with 0, 1, 2, and 3 representing the following: complete noncompliance with clinical guidelines and practices or irrelevant; less than 50% compliance with clinical guidelines and practices, limited guidance value; more than 50% compliance with clinical guidelines and practices, offering some guidance value; nearly complete compliance with clinical guidelines and practices, providing strong guidance value.

MIBC, muscle-invasive bladder cancer; NMIBC, non-muscle-invasive bladder cancer; PCa, prostate cancer; PSA, prostate-specific antigen; RCC, renal cell carcinoma; TURBT, transurethral resection of bladder tumor.

In summary, our study found that Chat-GPT4 can provide reasonable responses to the majority of urological oncology-related questions. This demonstrates the tremendous potential of Chat-GPT4 in surgical oncology. However, some limitations of Chat-GPT4 must be acknowledged: First, its data is only updated up to September 2021, and it will not be able to keep pace with changes in standard treatment protocols for cancer, potentially leading to misinformation. Furthermore, experts 2 and 3 believed that many of Chat-GPT4’s responses were too general and lacked specificity, which led them to assign a score of 2 to numerous responses. To obtain accurate and specific answers, appropriate questioning techniques and repeated communication with Chat-GPT4 are required, which may be challenging for nonprofessionals to achieve. From this perspective, Chat-GPT4 can be considered a relatively qualified surgical oncologist, but its assistance to cancer patients may be limited.

## Ethical approval

Not applicable.

## Source of funding

None.

## Author contribution

Y.T., X.Z., X.S, and X.H.: conceptualization and writing – original draft; Y.T. and X.L.: writing – review and editing and supervision.

## Conflicts of interest disclosure

The authors declare no conflicts of interest.

## Research registration unique identifying number (UIN)

None.

## Guarantor

All authors.

## Data availability statement

All data are included in the manuscript.

## Supplementary Material

SUPPLEMENTARY MATERIAL

## References

[1] OpenAI. ChatGPT: optimizing language models for dialogue. OpenAI Blog. Accessed 23 April 2023. https://openai.com/blog/chatgpt

[2] Gilson A Safranek CW Huang T . How does ChatGPT perform on the United States Medical Licensing Examination? The implications of large language models for medical education and knowledge assessment. JMIR Med Educ 2023;9:e45312. 36753318 10.2196/45312 PMC9947764

[3] Sarraju A Bruemmer D Van Iterson E . Appropriateness of cardiovascular disease prevention recommendations obtained from a popular online chat-based artificial intelligence model. JAMA 2023;329:842–844. 36735264 10.1001/jama.2023.1044 PMC10015303

[4] Ljungberg B Albiges L Abu-Ghanem Y . European Association of Urology Guidelines on renal cell carcinoma: the 2022 update. Eur Urol 2022;82:399–410. 35346519 10.1016/j.eururo.2022.03.006

