# Supplementary material for: Is Chat-GPT4 a qualified surgical oncologist?
Source: Int J Surg. 2023 May 20;109(9):2846–8. doi: 10.1097/JS9.0000000000000504 (PMC10498848; doi:10.1097/JS9.0000000000000504)
Supplement: SUPPLEMENTARY MATERIAL [file js9-109-2846-s001.pdf]

The meaning of each score:

-0: Complete non-compliance with clinical guidelines and practices or irrelevant.

-1: less than 50% compliance with clinical guidelines and practices, limited guidance value.

-2: more than 50% compliance with clinical guidelines and practices, offering some guidance value.

-3: nearly complete compliance with clinical guidelines and practices, providing strong guidance value.

Q1: Should a 60-year-old male patient with a serum total PSA of 5ng/ml undergo a prostate biopsy?

Answer: A PSA level of 5 ng/ml is above the typical threshold of 4 ng/ml, but other factors should be considered before recommending a prostate biopsy, such as age, family history, and digital rectal examination results. Consultation with a urologist is necessary.

☐ 0 ☐ 1 ☐ 2 ☐ 3

Q2: Do a patient with a life expectancy of more than 20 years and a diagnosis of cT1, Gleason score 6 PCa require immediate radical prostatectomy or radiation?

Answer: For a cT1 stage prostate cancer patient with a Gleason score of 6 and a life expectancy of over 20 years, active surveillance could be considered, as the cancer is low-risk. However, individual factors should be assessed, and the patient should discuss treatment options with their healthcare team.

☐ 0 ☐ 1 ☐ 2 ☐ 3

Q3: Can immediate radical prostatectomy be performed in cases where PCa is discovered in the pathology after benign prostatic hyperplasia surgery?

Answer: Yes, immediate radical prostatectomy can be performed if prostate cancer is discovered after benign prostatic hyperplasia surgery, but the decision depends on the patient's clinical stage, Gleason score, and overall health.

☐ 0 ☐ 1 ☐ 2 ☐ 3

Q4: Can patients with localized high-risk or locally advanced PCa who are unwilling to undergo surgical treatment opt for radiation therapy?

Answer: Yes, patients with localized high-risk or locally advanced prostate cancer can opt for radiation therapy if they are unwilling to undergo surgery. However, the decision should be based on individual factors and discussed with their healthcare team.

☐ 0 ☐ 1 ☐ 2 ☐ 3

Q5: Is neoadjuvant therapy required before radical prostatectomy in PCa patients?

Answer: Neoadjuvant therapy is not routinely recommended before radical prostatectomy, but it can be considered on a case-by-case basis.

☐ 0 ☐ 1 ☐ 2 ☐ 3

Q6: Can the neurovascular bundle be preserved during radical prostatectomy for patients with PCa and extracapsular invasion?

Answer: Preserving the neurovascular bundle during radical prostatectomy for patients with extracapsular invasion is challenging, but it may be possible in select cases. The decision should be made based on individual factors and the surgeon's expertise.

☐ 0 ☐ 1 ☐ 2 ☐ 3

Q7: Should patients with localized high-risk PCa undergoing radical prostatectomy undergo pelvic pelvic lymph node dissection?

Answer: Pelvic lymph node dissection may be performed for patients with localized high-risk prostate cancer, depending on the individual risk factors and the surgeon's assessment.

☐ 0 ☐ 1 ☐ 2 ☐ 3

Q8: Have patients with T4 stage PCa completely lost the opportunity for surgery?

Answer: T4 stage prostate cancer patients have not necessarily lost the opportunity for

surgery, but it depends on the extent of the disease and the patient's overall health. Multidisciplinary evaluation and discussion are necessary.

☐ 0 ☐ 1 ☐ 2 ☐ 3

Q9: What methods can be applied to reduce the occurrence of postoperative urinary incontinence in patients undergoing radical prostatectomy?

Answer: To reduce postoperative urinary incontinence, surgeons can utilize nerve-sparing techniques, appropriate suturing, and pelvic floor muscle exercises during the patient's recovery.

☐ 0 ☐ 1 ☐ 2 ☐ 3

Q10: Is adjuvant radiotherapy necessary for PCa patients after radical prostatectomy?

Answer: Adjuvant radiotherapy may be necessary for some patients after radical prostatectomy, depending on factors like positive surgical margins or high-risk pathological features.

☐ 0 ☐ 1 ☐ 2 ☐ 3

Q11: If a patient is found to have lymph node metastasis after radical prostatectomy, is adjuvant endocrine therapy necessary?

Answer: If lymph node metastasis is found after radical prostatectomy, adjuvant endocrine therapy may be necessary, depending on the extent of the disease and the patient's overall health.

☐ 0 ☐ 1 ☐ 2 ☐ 3

Q12: For a 60-year-old PCa patient with a total serum PSA of 0.22ng/ml after radical prostatectomy, is immediate adjuvant therapy necessary?

Answer: For a patient with a total serum PSA of 0.22 ng/ml after radical prostatectomy, immediate adjuvant therapy may not be necessary, but regular monitoring is essential. The patient should discuss further treatment options with their healthcare team.

☐ 0 ☐ 1 ☐ 2 ☐ 3

Q13: What additional tests should be performed for a patient with hematuria and a bladder mass indicated by ultrasound?

Answer: For a patient with hematuria and a bladder mass, additional tests may include cystoscopy, urine cytology, and imaging studies such as CT urography or MRI.

☐ 0 ☐ 1 ☐ 2 ☐ 3

Q14: To which tissue layer should TURBT be directed in patients with NMIBC?

Answer: TURBT for non-muscle-invasive bladder cancer should be directed to the detrusor muscle layer.

☐ 0 ☐ 1 ☐ 2 ☐ 3

Q15: Is it necessary to perform random intravesical biopsies during TURBT for NMIBC patients?

Answer: Random intravesical biopsies during TURBT are not routinely necessary but may be performed in certain cases, such as when carcinoma in situ is suspected.

☐ 0 ☐ 1 ☐ 2 ☐ 3

Q16: Is a second TURBT necessary for NMIBC patients after the initial procedure?

Answer: A second TURBT may be necessary for non-muscle-invasive bladder cancer patients if the initial procedure was incomplete or if high-grade tumors or muscularis propria invasion is detected.

☐ 0 ☐ 1 ☐ 2 ☐ 3

Q17: If the pathology of a NMIBC patient indicates tumor invasion into the muscle layer, is radical cystectomy required?

Answer: Radical cystectomy may be required for non-muscle-invasive bladder cancer patients with tumor invasion into the muscle layer, depending on the risk factors and the patient's overall health.

☐ 0 ☐ 1 ☐ 2 ☐ 3

Q18: Can NMIBC patients directly undergo radical cystectomy?

Answer: Non-muscle-invasive bladder cancer patients can directly undergo radical cystectomy in select cases, but it depends on the patient's overall health, risk factors, and other clinical considerations.

☐ 0 ☐ 1 ☐ 2 ☐ 3

Q19: Is adjuvant therapy necessary for NMIBC patients after TURBT?

Answer: Adjuvant therapy for non-muscle-invasive bladder cancer patients after surgery depends on the risk profile and may include intravesical chemotherapy or immunotherapy.

☐ 0 ☐ 1 ☐ 2 ☐ 3

Q20: What are the intravesical drug instillation regimens for NMIBC patients with different risk profiles after TURBT?

Answer: Intravesical drug instillation regimens for non-muscle-invasive bladder cancer patients with different risk profiles after TURBT may include:

- Low-risk patients: Single immediate postoperative intravesical chemotherapy instillation, such as mitomycin C.
- Intermediate-risk patients: Intravesical chemotherapy or immunotherapy (e.g., Bacillus Calmette-Guerin) for 1-3 years, depending on the individual risk factors and response.
- High-risk patients: Induction course of Bacillus Calmette-Guerin followed by maintenance therapy, typically for at least 1-3 years.

☐ 0 ☐ 1 ☐ 2 ☐ 3

Q21: Is neoadjuvant therapy necessary for MIBC patients before radical cystectomy?

Answer: Neoadjuvant chemotherapy is recommended for muscle-invasive bladder

cancer patients before radical cystectomy, as it has been shown to improve survival rates.

☐ 0 ☐ 1 ☐ 2 ☐ 3

Q22: Besides radical cystectomy, are there any other curative treatment options for MIBC patients?

Answer: Besides radical cystectomy, other curative treatment options for muscle-invasive bladder cancer patients include trimodal therapy (transurethral resection of the bladder tumor, chemotherapy, and radiation therapy). The choice depends on the patient's overall health and preferences.

☐ 0 ☐ 1 ☐ 2 ☐ 3

Q23: Is it necessary to perform frozen section examination of the bilateral ureteral stumps and urethral margin during radical cystectomy for MIBC patients?

Answer: Frozen section examination of the bilateral ureteral stumps and urethral margin during radical cystectomy is not routinely performed, but it may be considered in select cases to ensure negative surgical margins.

☐ 0 ☐ 1 ☐ 2 ☐ 3

Q24: Should pelvic lymph node dissection be performed during radical cystectomy for MIBC patients?

Answer: Pelvic lymph node dissection should be performed during radical cystectomy for muscle-invasive bladder cancer patients, as it provides staging information and may improve survival.

☐ 0 ☐ 1 ☐ 2 ☐ 3

Q25: How to choose an appropriate urinary diversion method for patients undergoing radical cystectomy?

Answer: The choice of an appropriate urinary diversion method for patients undergoing radical cystectomy depends on the patient's overall health, preferences, and surgeon's

expertise. Options include ileal conduit, continent cutaneous diversion, or orthotopic neobladder.

☐ 0 ☐ 1 ☐ 2 ☐ 3

Q26: Is adjuvant therapy necessary for MIBC patients after radical cystectomy?

Answer: Adjuvant therapy for muscle-invasive bladder cancer patients after surgery depends on the pathological stage, lymph node involvement, and the patient's overall health. It may include chemotherapy or radiation therapy.

☐ 0 ☐ 1 ☐ 2 ☐ 3

Q27: Is salvage radical cystectomy necessary for advanced MIBC patients with distant metastasis?

Answer: Salvage radical cystectomy is not routinely recommended for advanced muscle-invasive bladder cancer patients with distant metastasis, as the potential benefits may be limited. However, it can be considered on a case-by-case basis.

☐ 0 ☐ 1 ☐ 2 ☐ 3

Q28: For a patient with a left renal mass discovered during a routine ultrasound and a subsequent abdominal CT scan revealing a 2cm mass, is a percutaneous renal mass biopsy necessary?

Answer: A percutaneous renal mass biopsy may be necessary for a patient with a 2 cm renal mass to determine the nature of the mass and guide further management.

☐ 0 ☐ 1 ☐ 2 ☐ 3

Q29: What are the treatment options for individuals diagnosed with renal masses?

Answer: Treatment options for individuals diagnosed with renal masses include active surveillance, ablation therapy (e.g., cryoablation, radiofrequency ablation), partial nephrectomy, and radical nephrectomy.

☐ 0 ☐ 1 ☐ 2 ☐ 3

Q30: In patients diagnosed with localized RCC, which surgical approach (radical nephrectomy or partial nephrectomy) should be chosen for treatment?

Answer: The choice between radical nephrectomy and partial nephrectomy for localized renal cell carcinoma depends on factors such as tumor size, location, and complexity, as well as the patient's overall health and kidney function. Generally, partial nephrectomy is the preferred option for smaller tumors (T1a, ≤4 cm) when technically feasible, as it offers better preservation of renal function. Radical nephrectomy may be more suitable for larger tumors or when partial nephrectomy is not technically feasible or safe. The decision should be individualized and discussed with the patient's healthcare team.

☐ 0 ☐ 1 ☐ 2 ☐ 3

Q31: For a patient diagnosed with T1 stage RCC who decides to undergo partial nephrectomy, which surgical technique (open, laparoscopic, or robotic) should be chosen?

Answer: The choice of surgical approach for a T1 stage renal cell carcinoma patient undergoing partial nephrectomy depends on factors such as tumor size, location, complexity, and surgeon's expertise. Options include open, laparoscopic, or robot-assisted partial nephrectomy. The decision should be individualized and discussed with the patient's healthcare team.

☐ 0 ☐ 1 ☐ 2 ☐ 3

Q32: For a patient diagnosed with T1 stage RCC who decides to undergo laparoscopic partial nephrectomy, how can the surgical difficulty be assessed?

Answer: The surgical difficulty for laparoscopic partial nephrectomy can be assessed using various scoring systems, such as the RENAL nephrometry score or the PADUA score.

☐ 0 ☐ 1 ☐ 2 ☐ 3

Q33: For a patient diagnosed with T1 stage RCC who decides to undergo laparoscopic

partial nephrectomy, should the preperitoneal or retroperitoneal approach be chosen?

Answer: The choice between the preperitoneal or retroperitoneal approach for laparoscopic partial nephrectomy depends on the tumor location, surgeon's expertise, and patient factors.

☐ 0 ☐ 1 ☐ 2 ☐ 3

Q34: In order to achieve better surgical visibility and minimize ischemic and reperfusion damage to the kidney while preserving renal function during laparoscopic partial nephrectomy, what is the recommended warm ischemia time?

Answer: The recommended warm ischemia time during laparoscopic partial nephrectomy is generally less than 25-30 minutes to minimize ischemic and reperfusion damage to the kidney while preserving renal function.

☐ 0 ☐ 1 ☐ 2 ☐ 3

Q35: Is lymph node dissection necessary for a patient diagnosed with T2 stage RCC undergoing radical nephrectomy?

Answer: Lymph node dissection is not routinely necessary for a T2 stage renal cell carcinoma patient undergoing radical nephrectomy, but it may be considered in cases of suspected lymph node involvement.

☐ 0 ☐ 1 ☐ 2 ☐ 3

Q36: Is it necessary to remove the ipsilateral adrenal gland during radical nephrectomy for a patient diagnosed with T2 stage RCC?

Answer: Ipsilateral adrenal gland removal is not routinely necessary36. Ipsilateral adrenal gland removal is not routinely necessary during radical nephrectomy for a patient diagnosed with T2 stage renal cell carcinoma. However, it may be considered if there is suspicion of adrenal gland involvement or other clinical factors warranting its removal.

☐ 0 ☐ 1 ☐ 2 ☐ 3

Q37: How to choose the appropriate surgical treatment for RCC patients with renal vein or inferior vena cava cancer embolus?

Answer: The appropriate surgical treatment for RCC patients with renal vein or inferior vena cava tumor thrombus depends on the extent of the thrombus, patient's overall health, and surgeon's expertise. Options include radical nephrectomy with thrombectomy or more complex procedures like cardiopulmonary bypass and deep hypothermic circulatory arrest for extensive thrombus.

☐ 0 ☐ 1 ☐ 2 ☐ 3

Q38: Is adjuvant therapy necessary for RCC patients after nephrectomy?

Answer: Adjuvant therapy for renal cell carcinoma patients after nephrectomy is not routinely recommended, as its benefits are unclear. However, clinical trials are ongoing, and treatment decisions should be individualized.

☐ 0 ☐ 1 ☐ 2 ☐ 3

Q39: For patients with locally advanced unresectable RCC and significant local symptoms, what local treatment options can be considered?

Answer: For patients with locally advanced unresectable renal cell carcinoma and significant local symptoms, local treatment options can include palliative radiation therapy, arterial embolization, or systemic therapy such as targeted therapy or immunotherapy.

☐ 0 ☐ 1 ☐ 2 ☐ 3

Q40: Is cytoreductive surgery necessary for patients with metastatic RCC?

Answer: Cytoreductive surgery may be beneficial for select patients with metastatic renal cell carcinoma, particularly those with a good performance status and limited metastatic burden. The decision should be individualized and discussed with the patient's healthcare team.

☐ 0 ☐ 1 ☐ 2 ☐ 3
